# Monitoring the elimination of human African trypanosomiasis: Update to 2016

**DOI:** 10.1371/journal.pntd.0006890

**Published:** 2018-12-06

**Authors:** José R. Franco, Giuliano Cecchi, Gerardo Priotto, Massimo Paone, Abdoulaye Diarra, Lise Grout, Pere P. Simarro, Weining Zhao, Daniel Argaw

**Affiliations:** 1 World Health Organization, Control of Neglected Tropical Diseases, Innovative and Intensified Disease Management, Geneva, Switzerland; 2 Food and Agriculture Organization of the United Nations, Sub-regional Office for Eastern Africa, Addis Ababa, Ethiopia; 3 Food and Agriculture Organization of the United Nations, Animal Production and Health Division, Rome, Italy; 4 World Health Organization, Regional Office for Africa, Communicable Disease Unit, Brazzaville, Congo; 5 Consultant World Health Organization, Control of Neglected Tropical Diseases, Innovative and Intensified Disease Management, Geneva, Switzerland; Institute of Tropical Medicine, BELGIUM

## Abstract

**Background:**

Human African trypanosomiasis (HAT) is a neglected tropical disease targeted for elimination ‘as a public health problem’ by 2020. The indicators to monitor progress towards the target are based on the number of reported cases, the related areas and populations exposed at various levels of risk, and the coverage of surveillance activities. Based on data provided by the National Sleeping Sickness Control Programmes (NSSCP), Non-Governmental Organizations (NGOs) and research institutions—and assembled in the Atlas of HAT—the World Health Organization (WHO) provides here an update to 2016 for these indicators, as well as an analysis of the epidemiological situation.

**Results:**

Trends for the two primary indicators of elimination are on track for the 2020 goal: 2,164 cases of HAT were reported in 2016 (as compared to the milestone of 4,000 cases), and for the period 2012–2016 280,000 km^2^ are estimated to be at moderate risk or higher (i.e. ≥ 1 case/10,000 people/year), as compared to the milestone of 230,000 km^2^. These figures correspond to reductions of 92% and 61% as compared to the respective baselines (i.e. 26,550 HAT cases in the year 2000, and 709,000 km^2^ exposed at various levels of risk for the period 2000–2004). Among the secondary indicators, an overall improvement in the coverage of at risk populations by surveillance activities was observed. Regarding passive surveillance, the number of fixed health facilities providing gambiense HAT diagnosis or treatment expanded, with 1,338 enumerated in endemic countries in 2017 (+52% as compared to the survey completed only sixteen months earlier). Concerning rhodesiense HAT, 124 health facilities currently provide diagnosis or treatment. The broadening of passive surveillance is occurring in a context of fairly stable intensity of active case finding, with between 1.8 million and 2.4 million people screened per year over the period 2012–2016.

**Discussion:**

Elimination of HAT as a public health problem by 2020 seems within reach, as the epidemiological trends observed in previous years are confirmed in this latest 2016 monitoring update. However, looking beyond 2020, and in particular to the 2030 goal of elimination of transmission as zero cases for the gambiense form of the disease only, there is no room for complacency. Challenges still abound, including ensuring the effective integration of HAT control activities in the health system, sustaining the commitment of donors and HAT endemic countries, and clarifying the extent of the threat posed by cryptic reservoirs (e.g. human asymptomatic carriers and the possible animal reservoirs in gambiense HAT epidemiology). WHO provides through the network for HAT elimination the essential coordination of the wide range of stakeholders to ensure synergy of efforts.

## Author summary

Human African trypanosomiasis (HAT), also known as sleeping sickness, is a rare disease today, but throughout the 20^th^ century it has demonstrated its potential for devastating epidemics. Over the last twenty years, substantial efforts against HAT enabled to dramatically reduce its prevalence, and the disease is now targeted for elimination by the World Health Organization (WHO). In this paper we provide an update to 2016 of the epidemiological situation, focusing on the number of reported cases, the areas and populations exposed at various levels of risk, and the coverage and intensity of surveillance activities. The trends show that attaining the 2020 target of elimination as a public health problem is within reach, with 2,164 reported cases in 2016 (as compared to the targeted milestone for the same year of 4,000 cases), and with an improved coverage of at risk populations by surveillance activities. Beyond 2020, achieving elimination of transmission for the gambiense form of the disease (targeted for 2030) will be more challenging, and it will require the sustained commitment of HAT endemic countries and donors, the adoption of innovative disease control approaches, and the coordination of a broad range of stakeholders.

## Introduction

Since the beginning of the 21^st^ century, and following the reinforcement of control and surveillance activities against human African trypanosomiasis (HAT), the number of reported cases has been decreasing steadily [1, 2]. Control activities have been coordinated and implemented by National Sleeping Sickness Control Programmes (NSSCPs) with the support of the World Health Organization (WHO), in the framework of a longstanding public-private partnership with Sanofi and Bayer, and together with bilateral cooperation, non-governmental organizations (NGOs) and other stakeholders. In this context, in 2012 WHO included the goal of eliminating sleeping sickness as public health problem by 2020 in its Neglected Tropical Diseases (NTD) roadmap [3]. Beyond 2020, WHO, in coordination with NSSCPs, set the goal of elimination of HAT as interruption of transmission by 2030 (i.e. reaching and sustaining zero HAT cases) for the gambiense form of the disease only.

Global indicators and milestones were defined to monitor the progress towards elimination, that is presented every two years at WHO HAT stakeholders meetings [4–7]. Monitoring updates for 2012 and 2014 have been published [8, 9], which indicated that progress towards HAT elimination met the established milestones. The present paper provides a comprehensive review of the progress up to 2016.

## Materials and methods

### Ethics statement

The research does not directly involve human participants. No individual data is used in the paper. All the data used are provided routinely by NSSCPs, NGOs and research institutions as epidemiological information and are fully anonymized.

### Global indicators of HAT elimination

As established by a WHO Expert Committee on control and surveillance of HAT [1], subsequently refined by the WHO HAT elimination Technical Advisory Group (HAT-e-TAG), and finally endorsed by the WHO Neglected Tropical Diseases Scientific and Technical Advisory Group (NTD-STAG), HAT elimination is to be monitored through two primary indicators: (1) the annual number of reported cases, where the target for the year 2020 is fewer than 2,000 cases [1], and (2) the area at risk reporting ≥ 1 case/10,000 people/year (calculated over a 5-year period), where the target for 2020 (more exactly, for the 5-year period 2016–2020) is a reduction of 90% as compared to the 2000–2004 baseline [10, 11]. Three secondary indicators should also be followed: (a) the geographic distribution of the disease, (b) the population exposed at different levels of risk, and (c) the coverage of the exposed populations by control and surveillance activities [1].

### Methods

The methods used to estimate the global indicators of HAT elimination have already been described in previous biennial updates, most notably for 2012 [8] and 2014 [9]. Additional methodological details are available from more focused publications dealing with HAT geographic distribution [12, 13], HAT risk estimation [14, 15], HAT cases detected in non-endemic countries [16], and HAT population at risk potentially covered by fixed health facilities with capacities for HAT diagnosis and treatment [17]. To avoid repetition, in this paper only a few methodological updates are provided.

Concerning the number and geographic distribution of HAT cases reported annually, the number of people actively screened for gambiense HAT, as well as the areas and populations at risk of HAT, the present paper provides an update to 2016.

As regards the estimation of the population at risk potentially covered by fixed health facilities with capacities for HAT diagnosis and treatment, a survey of the facilities was carried out between March and June 2017. Subsequently, a time-distance analysis was performed to estimate their physical accessibility for the at-risk populations [17]. In this exercise, the HAT risk distribution for 2012–2016 was used for stratification of results.

The present paper includes data from the integrated passive surveillance system, which is being implemented in the context of HAT elimination, mainly in countries and areas where HAT prevalence became very low and systematic active screening is no longer cost-effective. The system turns around sentinel sites, which are selected following epidemiological criteria. Based on their respective capacities, sentinel sites perform different levels of diagnosis (clinical suspicion, serological suspicion and parasitological confirmation) on patients attending the facilities for any health problem. The HAT surveillance activity is fully integrated in the activities of the centres. Capacities for HAT diagnosis in these centres are upgraded through training, provision equipment and materials and supervision. Data are routinely collected and periodically analysed. Each HAT case detected triggers a response consisting of an active screening campaign (i.e. reactive screening) in the community of origin of the affected patient.

All data used in this epidemiological update are assembled in the Atlas of HAT [13], and they have been provided by the NSSCPs, as well as by NGOs and research institutions.

## Results

### Number of HAT cases reported annually

**Fig 1:**
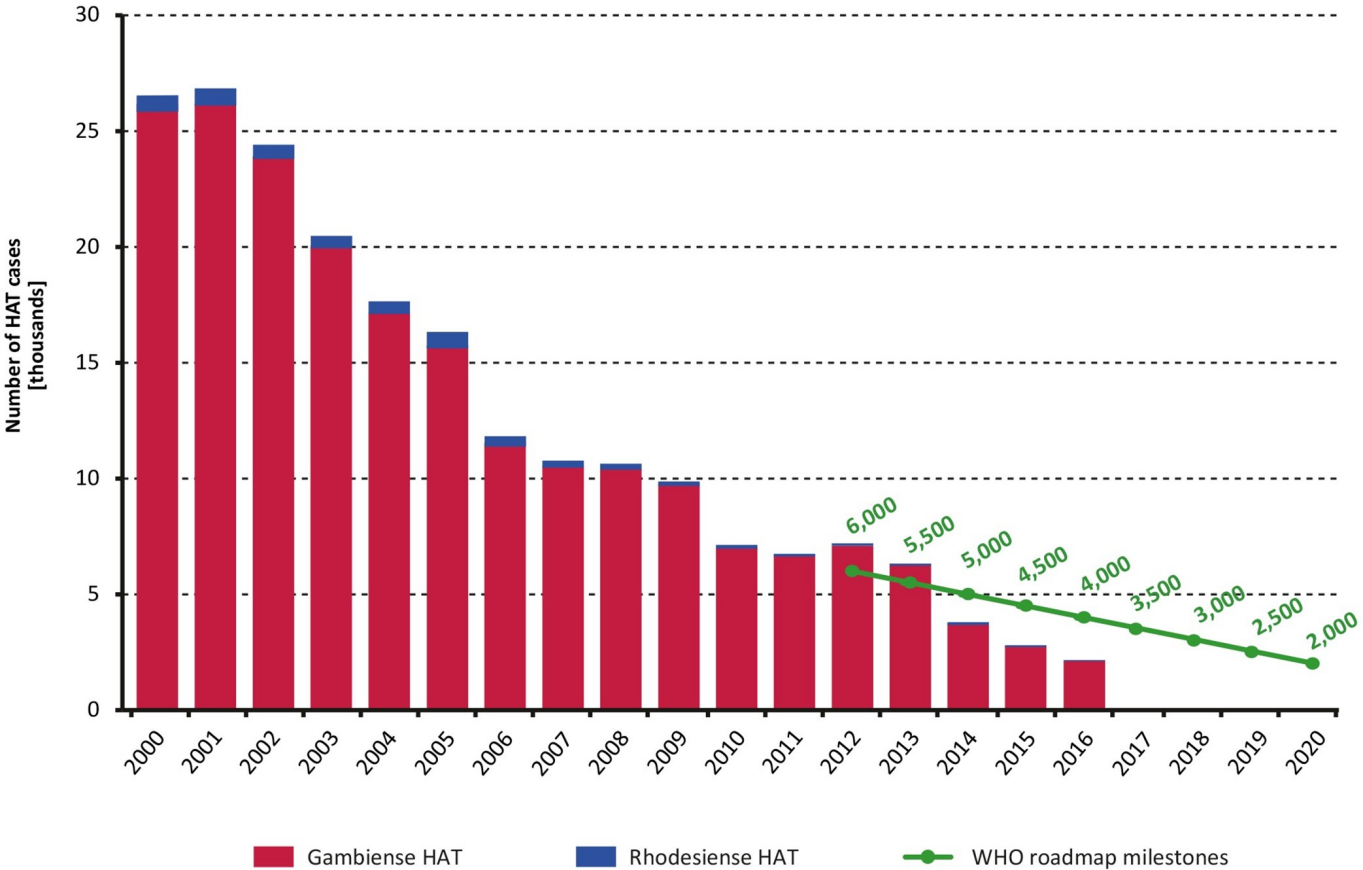
Total number of reported cases of HAT (gambiense and rhodesiense) per year (2000–2016). The green line shows the milestones set in the WHO Roadmap for HAT elimination [3].

The decreasing trend observed for this indicator in previous years has continued. In 2015, a total of 2,801 new HAT cases were reported and in 2016 the figure went down to 2,164, including both gambiense and rhodesiense HAT (Fig 1). Such record low figures are 1,699 and 1,836 cases below the respective milestones as set in the WHO roadmap. Details on the HAT cases reported by country and by year (period 2000–2016) are shown in Table 1 (*T*. *b*. *gambiense*) and Table 2 (*T*. *b*. *rhodesiense*).

**Table 1:** *T*. *b*. *gambiense* HAT: New cases reported between 2000 and 2016.

Country	2000	2001	2002	2003	2004	2005	2006	2007	2008	2009	2010	2011	2012	2013	2014	2015	2016	Total
Angola	4,546	4,577	3,621	3,115	2,280	1,727	1,105	648	517	247	211	154	70	69	36	35	20	22,978
Burkina Faso	0	0	0	0	0	0	0	0	0	0	0	0	0	0	0	1	0	1
Cameroon	27	14	32	33	17	3	15	7	13	24	16	15	7	6	7	6	6	248
Central African Republic	988	718	572	539	738	666	460	654	1,194	1,054	395	132	381	59	194	147	101	8,992
Chad	153	138	715	222	483	190	276	97	196	510	232	276	197	195	95	67	54	4,096
Congo	111	894	1,005	717	873	398	300	189	182	87	87	61	39	20	21	36	18	5,038
Côte d’Ivoire	188	92	97	68	74	42	29	13	14	8	8	10	9	7	6	3	0	668
Democratic Republic of the Congo	16,951	17,300	13,816	11,459	10,339	10,249	8,013	8,155	7,318	7,178	5,624	5,590	5,969	5,649	3,205	2,347	1,768	140,930
Equatorial Guinea	16	17	32	23	22	17	13	15	11	7	8	1	2	3	0	0	3	190
Gabon	45	30	26	26	49	53	31	30	24	14	22	17	9	17	10	9	10	422
Ghana	1	0	0	0	0	0	0	0	0	0	0	0	0	1	0	0	0	2
Guinea	52	72	132	130	95	94	48	69	90	79	68	57	70	78	33	29	108	1,304
Nigeria	14	14	26	31	10	21	3	0	0	0	2	3	2	0	0	0	1	127
South Sudan	1,801	1,919	3,121	3,061	1,742	1,853	789	469	623	373	199	272	317	117	63	45	17	16,781
Uganda	948	310	604	517	378	311	290	120	198	99	101	44	20	9	9	4	4	3,966
Total	25,841	26,095	23,799	19,941	17,100	15,624	11,372	10,466	10,380	9,680	6,973	6,632	7,092	6,230	3,679	2,729	2,110	205,743

Other historically *T. b. gambiense* HAT endemic countries not reporting cases but with surveillance activities are Benin, Mali, Niger, Senegal, Sierra Leone, and Togo. In the Gambia, Guinea Bissau and Liberia no cases are reported but no surveillance activity is known.

**Table 2:** *T*. *b*. *rhodesiense* HAT: New cases reported between 2000 and 2016.

Country	2000	2001	2002	2003	2004	2005	2006	2007	2008	2009	2010	2011	2012	2013	2014	2015	2016	Total
Kenya	15	10	11	0	0	0	1	0	0	1	0	0	2	0	0	0	0	40
Malawi	35	38	43	70	48	41	58	50	49	39	29	23	18	35	32	30	35	673
Mozambique	-	-	1	-	1	-	-	-	-	-	-	-	-	-	-	-	-	2
Uganda	300	426	327	338	335	473	261	119	138	129	112	84	71	43	70	28	10	3,264
United Republic of Tanzania	350	277	229	113	159	185	127	126	59	14	5	1	4	2	1	2	4	1,658
Zambia	9	4	5	15	9	7	6	10	13	4	8	3	6	6	12	9	4	130
Zimbabwe	-	-	-	-	-	3	-	-	0	3	2	4	9	1	3	3	1	29
Total	709	755	616	536	552	709	453	305	259	190	156	115	110	87	118	72	54	5,796

Other historically *T. b. rhodesiense* HAT endemic countries not reporting cases are Burundi, Ethiopia and Rwanda. Botswana, Namibia and Swaziland are considered free of the vector for the transmission of T. b. rhodesiense HAT.

Most of the reported cases correspond to gambiense HAT, with 2,729 and 2,110 cases in 2015 and 2016 respectively—the 2016 figure corresponding to a 92% reduction compared to 2000. The heaviest burden of gambiense HAT continues to be carried by the Democratic Republic of the Congo (DRC), which in 2016 accounted for 1,768 cases (84% of the gambiense HAT burden).

**Fig 2:**
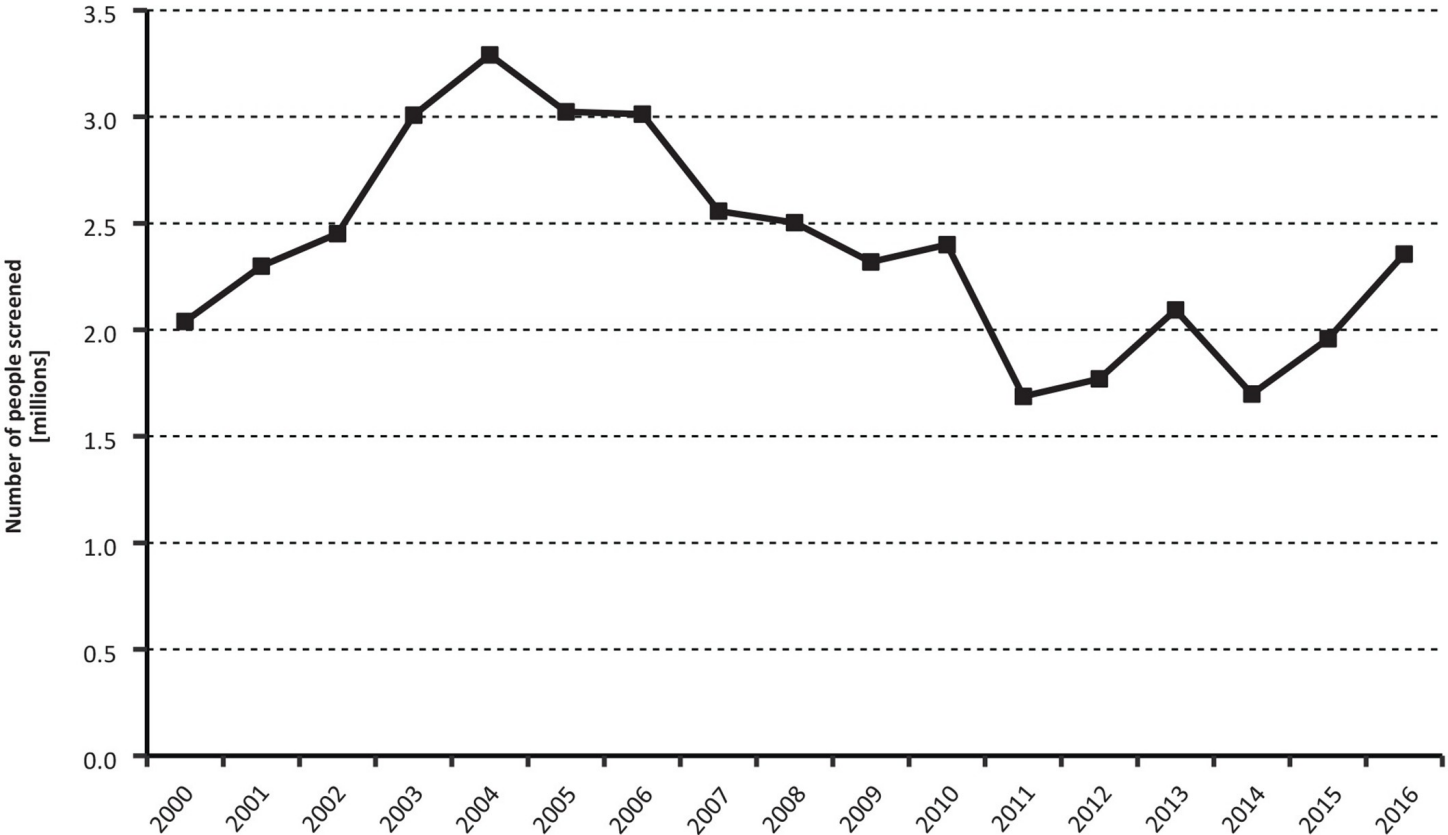
Number of people screened by active case-finding surveys, in countries endemic for *T*. *b*. *gambiense* (2000–2016). Concerning rhodesiense HAT, 72 and 54 cases were reported in 2015 and 2016 respectively, which constitute 2.5% of the total HAT reported cases. The cases reported in 2016 represent a reduction of 92.4% compared to the year 2000.

Active case finding has been one of the major tools to combat *T*. *b*. *gambiense* transmission. The number of people actively screened in the period 2000–2016 is presented in Fig 2. Despite year-to-year variations, the overall intensity of active case finding has been fairly stable over the described time period, and it has varied between 1.8 million and 2.4 million people screened per year over the last five-year period (2012–2016). However, whereas in the year 2000 one HAT case was detected for every 152 people actively screened (13,400 cases actively detected / 2.039,254 people screened), in 2016 1,981 people needed to be screened per each case actively detected (1,208 cases actively detected / 2,355,885 people screened). In addition to active screening, it is worth noting that the number of cases passively detected correspond to 49% of the gambiense HAT cases reported from 2000, varying from 39.5% in 2000 to 44% in 2016, with a maximum of 59% in 2007.

### Geographic distribution of HAT

**Fig 3:**
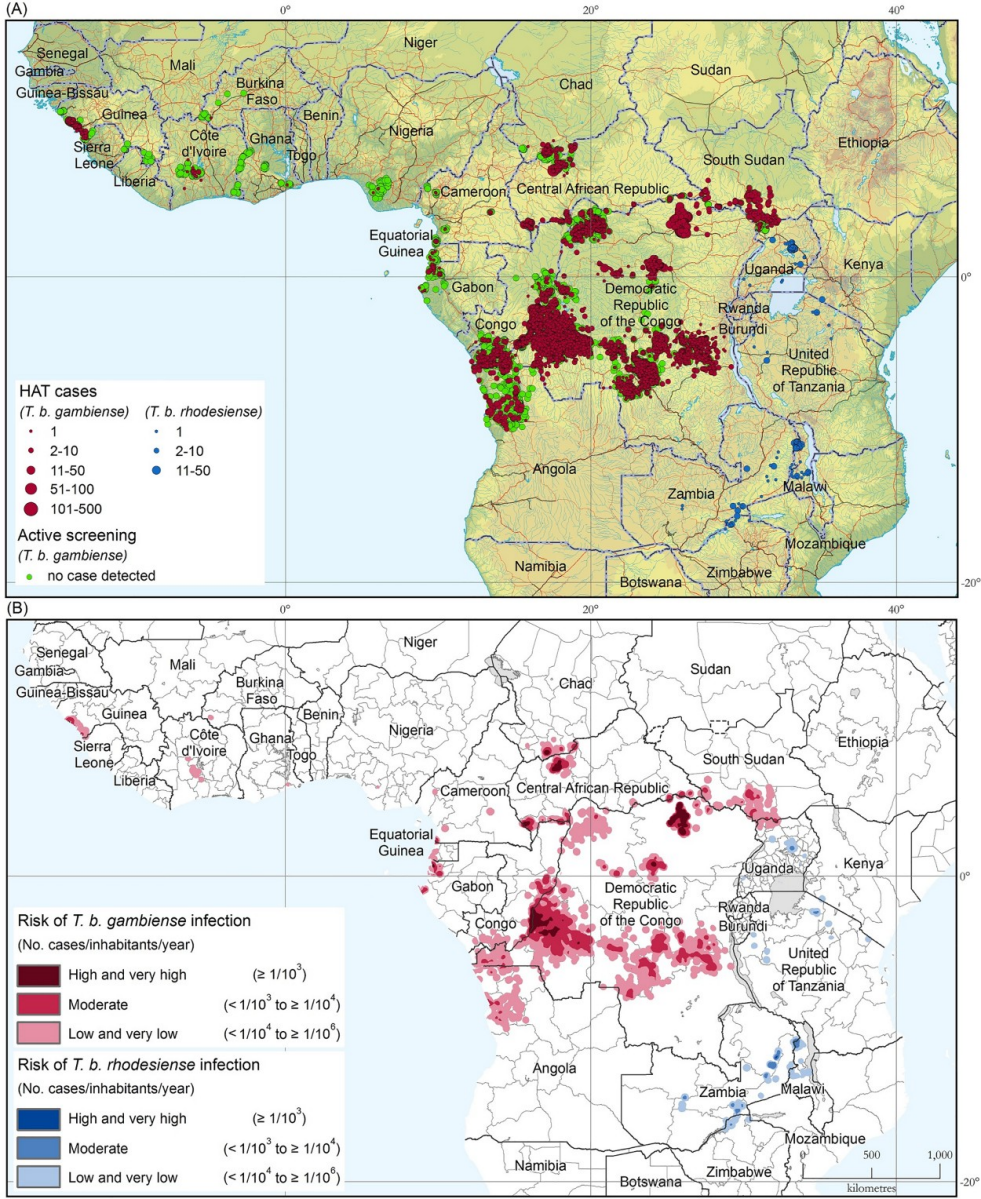
(a) The distribution of human African trypanosomiasis. Period 2012–2016. (b) The areas at risk of HAT infection. Period 2012–2016. The maps were created with ArcGIS 10.0 software (http://desktop.arcgis.com/). The base layers include the Shuttle Radar Topography Mission (https://www2.jpl.nasa.gov/srtm/), the Vector Map Level 0 (http://earth-info.nga.mil/publications/vmap0.html), and the Global Administrative Unit Layers (http://www.fao.org/geonetwork/srv/en/main.home).

Fig 3A shows the geographic distribution of sleeping sickness cases for the 5-year period 2012–2016. The locations of active screening activities where no cases were detected are included (green dots).

For the period 2012–2016, 22,281 new HAT cases were reported, 87.3% of which could be mapped at the village level. For the remaining 12.7% of the cases, village-level mapping was not possible and mapping was done at the district or sub-district level. For the whole period 2000–2016 (2000 being the start year of the Atlas of HAT), a total number of 211,539 cases has been included in the database. Of these, 93.2% cases have been mapped at the village level, for a total of 31,115 mapped villages. The average accuracy for mapped HAT cases is presently estimated at 1.4 km, and it is being continuously improved.

#### Gambiense HAT

In West Africa, Guinea continues to be the most affected country. Control and surveillance activities returned in 2016 to a normal pace after the Ebola outbreak, and as a result, the number of reported cases increased [18]. In Côte d’Ivoire the number of cases per year remains below 5, despite control and surveillance activities having been reinforced. One autochthonous case was detected in Burkina Faso in 2015 by the integrated passive surveillance system [19]. This case occurred more than 15 years after the last autochthonous case in the country. Clinical, serological and parasitological features were typical of *T*. *b*. *gambiense*, and only the molecular biology investigations failed to confirm the subspecies. Epidemiological investigations strongly suggested local transmission, possibly connected to transmission areas in Côte d’Ivoire through travel and migrations of community and family members. The reactive screening carried out in the area did not detect any additional case in humans, nor a potential animal reservoir.

In Benin, Ghana, Mali, Nigeria and Togo no local cases have been detected, even though a surveillance system for HAT based on sentinel sites is operational. However, an exported case from Nigeria was reported in the United Kingdom [20], and an exported case (a Malian national) was reported in France [21], for whom infection most probably occurred during a migratory journey through HAT foci in costal Guinea. An assessment of the epidemiological situation was carried out in Guinea-Bissau and no cases were detected; however, favourable conditions for HAT transmission are present, and the establishment of a surveillance system is required.

In Central Africa, active screening activities have continued to be routinely carried out in Cameroon, Chad, Congo, Equatorial Guinea and Gabon, and the system of passive case detection has been reinforced. In Chad and Congo, a steadily decreasing trend has been observed, whilst in Cameroon, Equatorial Guinea and Gabon the number of reported cases has been stable at a low level. In all these countries, since active and passive screening is ongoing in the transmission areas, it is believed that the number of reported cases reflects the real disease trend. In Central African Republic (CAR) the number of cases reported annually is still over 100. However, data from CAR have to be interpreted with care because active screening has been erratic in the foci of Nola (Sangha-Mbaere) and Lobaye, and practically non-existent in the foci of Haut-Mbomou and Ouham, owing to important security constraints, and cases reported rely only on a weak passive surveillance.

In Uganda, the downward trend continued, with just 4 cases reported per year (period 2015–2016) in a context of reinforced passive surveillance. Nevertheless, an important number of refugees from South Sudan has recently settled in the West Nile Region, which could result in an increased risk of HAT transmission [22]. In South Sudan the reduction in reported cases (i.e. only 17 in 2016) has to be interpreted cautiously and it is actually a reason for concern, as it probably reflects the decrease in control and surveillance activities linked to social unrest rather than a real reduction.

In Angola the decreasing trend is marked (from 1,777 cases in 2007–2011 to 230 in 2012–2016). However, it must be noted that in the last two years, while passive detection capacities have been maintained, active screening has been significantly weaker than before.

The DRC continues to be the country with the heaviest burden of HAT [23], but nevertheless it is showing a steady decrease (from 53,876 cases in 2002–2006 to 33,865 in 2007–2011 and to 18,938 in 2012–2016). The reported trend is likely to reflect a real reduction of infections, as in recent years the intensity of active screening has been maintained with more than 2 million people screened per year, and passive surveillance has been strengthened. However, within this general context, control activities have been neglected in some transmission areas (e.g. Haut Uele), where there is a need to reinforce the coverage.

#### Rhodesiense HAT

The number of reported cases of rhodesiense HAT continues to decrease in the majority of the affected countries. Over the last four years Malawi has reported a fairly constant number of cases (around 30 per year). Since 2015 it is the country reporting the highest caseload, in a context of wildlife-related transmission, and with a maintained capacity for surveillance. An important decrease in reported cases has been observed in Uganda, where transmission is mainly related to cattle; multisectoral disease control activities in a One Health framework (i.e. including the veterinary dimension) are enabling to keep the transmission of the disease under control in the country.

Tanzania, Zambia and Zimbabwe continue to report low numbers of cases, but HAT surveillance in these countries has declined in recent years. It is noteworthy that 3 of the 6 cases reported in Tanzania in 2015–2016 were diagnosed in non-endemic countries among travellers returning from Tanzania. Looking at the 5-year period 2012–2016, the same pattern is observed in Kenya, Tanzania, and Zambia, with a remarkable proportion of cases reported in non-endemic countries. In Kenya, no autochthonous case has been reported over the last 5 years, despite an operational surveillance system. The last two cases reported in 2012 were tourists infected during visits to the Masai-Mara National Reserve, and diagnosed in Germany and Belgium. In Tanzania, out of 13 cases declared in this period, 5 cases were diagnosed in tourists in non-endemic countries (Sweden, South Africa, Spain, Italy and Netherlands). In Zambia, out of a total of 37 cases declared, 9 were diagnosed among travellers in non-endemic countries (2 in South Africa, 2 in USA and 1 respectively in France, Argentina, United Kingdom, India and Canada).

In recent years, with the exception of cattle-related transmission areas in south-east Uganda, the spatial pattern of distribution of rhodesiense HAT appears closely linked to wildlife protected areas. In particular, cases have been associated to Murchinson Falls and Queen Elizabeth National Parks (Uganda), Ugalla Muyowosi Game Reserve, Katavi National Park, Mkomazi National Park, Ngorongoro Conservation Area, Serengeti, Lake Manyara and Tarangire National Parks (Tanzania), Vwaza Marsh and Nkhotakota Wildlife Reserves and Kasungu National Park (Malawi), Kafue, Lower Zambezi, North and South Lwanga, Luambo and Lukusuzi National Parks (Zambia) and Mana Pool National Park (Zimbabwe).

### Areas and population at risk of HAT

#### Areas at risk of HAT

**Fig 4:**
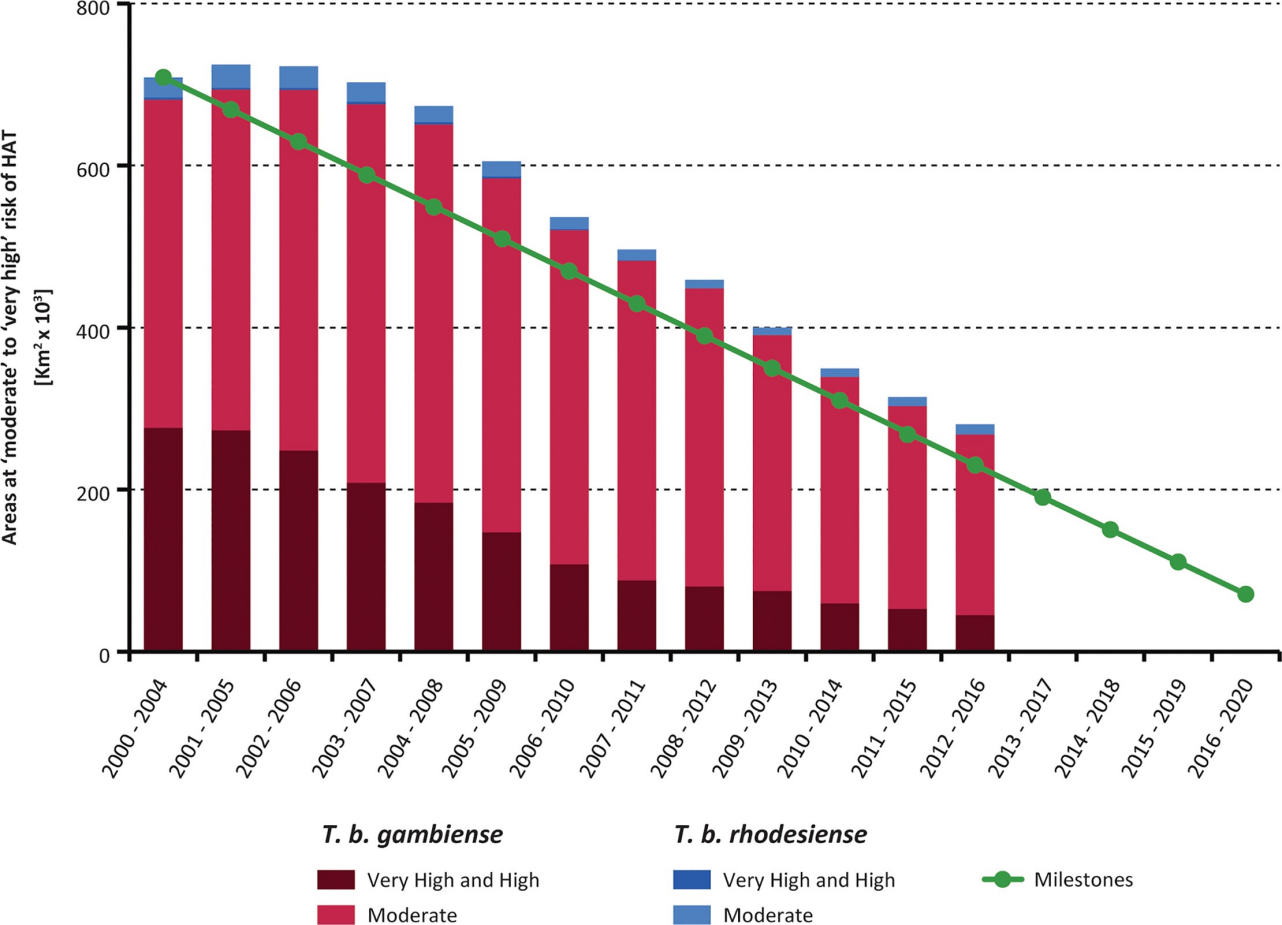
Trends in areas at risk of gambiense and rhodesiense HAT where the disease is still considered as a public health problem (2000–2004 to 2012–2016). The green line shows the milestones set by the WHO-NTD-STAG to achieve thfood e elimination of HAT as a public health problem by 2020.

Fig 3B shows the areas at different levels of HAT risk for the 5-year period 2012–2016. These areas are also summarized by country in S1 File. Fig 4 shows the evolution of the area reporting ≥ 1 case/ 10,000 inhabitants/year that is to say, where HAT risk is considered a public health problem (i.e. moderate, high and very high risk), and presented in five-year periods from 2000–2004 to 2012–2016; The area at very high, high or moderate risk for HAT has shrunk by 428,000 km^2^ from the 709,000 km^2^ at the baseline period (2000–2004), corresponding to a 61% reduction. The milestones to attain the 2020 target are represented by the green line (i.e. a reduction of 90% as compared to the 2000–2004 baseline).

For gambiense HAT, 0.99 million square kilometres are estimated to be at various levels of risk (period 2012–2016). The area where HAT is still considered a public health problem is represented by almost 45,000 km^2^ at very high and high risk, and almost 223,000 km^2^ at moderate risk (Table A in S1 File). These figures, as compared to the 2000–2004 baseline, indicate a reduction of 60% for the area where gambiense HAT is considered a public health problem. A reduction of area at risk was observed in all countries, each one contributing to the overall reduction to a variable extent.

For rhodesiense HAT, approximately 90,000 km^2^ are estimated to be the area of infection risk (Table B in S1 File). Most of this area is in the low and very low risk categories (79,000 km^2^), where HAT is not considered a public health problem. Only 12,500 km^2^ are at moderate risk or higher in the period 2012–2016; this represents a decrease of 55% of the area with ≥ 1 case/10,000 inhabitants/year as compared to the 2000–2004 baseline, but an increase of 2,200 km^2^ (+21.3%) as compared to the period 2010–2014. The increase is mainly linked to the area at risk in Zambia, which in turn is related to the presence of several exported cases (tourists) infected in conservation areas with very low population density.

#### Population at risk of HAT

In total, 57 million people are estimated to live at various levels of risk of sleeping sickness (period 2012–2016).

For gambiense HAT, 53 million people are estimated to be at risk of infection. Out of these, 8.2 million people live in areas where gambiense HAT is still considered a public health problem (in particular, less than one million people–i.e. seven hundred fifty thousand—are at very high and high risk, and 7.5 million are at moderate risk (Table A in S2 File).

For rhodesiense HAT, over 4 million people are estimated to be at risk (Table B in S2 File). Most (93%) are in the low and very low risk categories, while only less than 300,000 people are living in areas where sleeping sickness is considered as a public health problem.

### Population at risk potentially covered by fixed health facilities with capacities for HAT diagnosis and treatment

#### Survey and mapping of fixed health facilities

For gambiense HAT, the survey completed in June 2017 revealed the existence of 1,338 fixed health facilities with capacity for diagnosis or treatment (+52% as compared to the survey September 2015—February 2016 [9]). The complete results of the survey are provided in Table A in S3 File. Diagnosis is available in 1,246 facilities (+41%) and treatment in 642 (+24%). Fifty-four percent of the facilities (i.e. 723) are found in the DRC.

The capacity for parasitological diagnosis is available in 354 facilities (+9%), while the treatment for first-stage disease (pentamidine) is provided by 640 facilities (+24%), and for second-stage (NECT) is provided by 253 facilities (+12%), as this treatment requires higher capacities for its administration.

For rhodesiense HAT, 124 facilities (+12%) offer diagnosis in seven endemic countries, i.e. Kenya, Malawi, Rwanda, Uganda, United Republic of Tanzania, Zambia and Zimbabwe. All of these can perform clinical diagnosis, while parasitological diagnosis is offered by 76% of the facilities. Forty-four health facilities are involved in rhodesiense HAT treatment, and all provide both suramin for first-stage disease and melarsoprol for second-stage.

The geographic distribution of the health structures involved in HAT diagnosis and treatment is shown in S4 File.

The results of the survey completed in June 2017 show a consistent increase in the number of facilities providing all the different diagnostic and treatment services, with an overall improvement in the accessibility of diagnosis and treatment for HAT.

#### Population at risk potentially covered by fixed health facilities

Table 3 summarizes the potential coverage of the population at risk of HAT by fixed health facilities.

For the diagnosis of gambiense HAT, 58% of the at-risk population (31 million people) are within one hour travel of a competent facility, while 21% are at 1 to 3 hours and 10% at 3 to 5 hours travel (11 and 7 million respectively). For treatment, the corresponding figures are 23.5 (44%), 16.5 (31%), and 6 million (11%). These figures show a slight improvement as compared to the previous survey, when accessibility for the same categories was 52%, 25% and 11% for diagnosis and 42%, 31% and 13% for treatment [9], and hence an increased coverage of the at risk populations for both diagnosis and treatment.

**Table 3:** People at risk of HAT that are potentially covered by facilities with diagnostic and treatment capabilities for HAT.

Risk category	People at risk	People at risk covered by facilities with HAT capabilities											
		Diagnosis						Treatment					
		≤ 1-hour travel		≤ 3-hour travel		≤ 5-hour travel		≤ 1-hour travel		≤ 3-hour travel		≤ 5-hour travel	
	(no. persons × 10^3^)	(no. persons × 10^3^)	% of at risk	(no. persons × 10^3^)	% of at risk	(no. persons × 10^3^)	% of at risk	(no. persons × 10^3^)	% of at risk	(no. persons × 10^3^)	% of at risk	(no. persons × 10^3^)	% of at risk
**gambiense HAT**													
High and very high	750	327	44	468	62	538	72	350	47	471	63	537	72
Moderate	7,494	3,152	42	5,598	75	6,523	87	3,125	42	5,571	74	6,537	87
Low and very low	44,891	27,251	61	35,912	80	40,191	90	20,070	45	33,817	75	38,678	86
Total	53,134	30,730	58	41,979	79	47,252	89	23,545	44	39,859	75	45,752	86
**rhodesiense HAT**													
High and very high	-	-	-	-	-	-	-	-	-	-	-	-	-
Moderate	286	172	60	248	87	269	94	143	50	224	78	254	89
Low and very low	3,935	1,350	34	2,727	69	3,300	84	864	22	2,423	62	3,000	76
Total	4,222	1,522	36	2,975	70	3,569	85	1,007	24	2,647	63	3,254	77

It is noteworthy that among the population at highest risk of gambiense HAT (i.e. very high and high), over 200,000 people (28%) are at >5 hours travel from a competent diagnostic facility. This observation highlights one of the main challenges in HAT control and elimination: the persistence of transmission in insecurity-ridden and difficult-to-access areas. In fact, the bulk of the uncovered population can be found in North-Eastern DRC (*Province Orientale*) where the national programme has been unable to setup HAT services. An NGO that was active in the area for some years identified and partly addressed the problem, but it could not sustain its activities after the emergency was brought under control.

Among the population at risk of rhodesiense HAT, 1.5, 1.5, and 0.6 million people are respectively within one, one to three, and three to five hour travel of a competent diagnostic facility (corresponding to 36%, 34%, and 15% of the at-risk population). For treatment, the corresponding estimates are 1.0, 1.6, and 0.7 million (i.e. 24%, 39%, and 14% of the at-risk population). These coverage data, expressed as percentage of the at-risk population, are inferior to those of the previous survey. This is due to the fact that a part of the population that was formerly at risk, but which is no longer so was located in areas with good coverage (e.g. in Uganda); as a result, the bulk of the population at risk has shifted towards countries where health facilities are at a larger distance from the at-risk population.

It is worth noting that health facilities providing HAT diagnosis and treatment also cover a substantial number of people who are at marginal risk, including areas that were at risk of the disease in the past. In these areas there is an improvement in the accessibility to diagnostic facilities as a result of the expansion of the HAT surveillance system.

## Discussion

Data for the biennium 2015–2016 show that the epidemiological situation of HAT is following the trends observed in previous years (2000–2014), thus indicating further progress towards the goal of elimination of HAT as a public health problem by 2020. The number of cases reported has decreased by 92% since the year 2000 (from 26,550 to 2,164). The area reporting ≥ 1 case/10,000 inhabitants/year in the five-year period (2012–2016) has shrunk by 61% from the baseline period (2000–2004).

Concerning gambiense HAT, the decrease in the number of cases and in the area reporting ≥ 1 case/10,000 inhabitants/year is observed in a context of sustained active screening and reinforced passive surveillance in the majority of endemic countries. Importantly, the number of health facilities providing gambiense HAT diagnosis and treatment keeps increasing, and so does their potential coverage of the at-risk population. Therefore, it can be considered that the observed trends are very likely to reflect a real abatement in disease transmission, despite the challenges always posed by under-detection.

Despite the very encouraging global picture painted by the global indicators, the situation is not homogeneous, as gambiense HAT control and surveillance has weakened in a few countries, mainly owing to security constraints (e.g. Central African Republic and South Sudan). In addition, if the awareness of the disease wanes and there is an insufficient country ownership of the elimination process and goals, there could be a risk of deceleration of control and surveillance activities. The consequences of such deceleration have already been painfully experienced in the recent history of HAT.

Looking at the rhodesiense form of the disease, which continues to represent a small proportion of the total number of HAT reported cases, the decreasing trend seems to be sustained, with a further reduction in the number of cases (92% from 2000 to 2016, from 709 cases to 54), and the corresponding dwindling of the area at risk. Nevertheless, these reported trends are likely to be less representative of the real situation than those for gambiense HAT. In particular, disease surveillance has weakened in such countries as Tanzania, Uganda (for rhodesiense HAT), Zambia and Zimbabwe. The widespread adoption of serological rapid tests to diagnose malaria, which have replaced microscopic examination, now prevents the accidental diagnosis of rhodesiense HAT when testing for malaria. The problem is exacerbated by a concomitant decrease in HAT-skilled staff who could maintain knowledge and awareness of the disease. These factors, compounded by the acute clinical progression of rhodesiense HAT usually prevalent in remote rural areas, are likely to result in non-negligible under-detection. An indirect indication of this possible under-detection is the fact that 8 cases (6% of the total rhodesiense HAT caseload) were diagnosed in non-endemic countries among returning tourists in 2015–2016.

## Conclusions

Progress in the indicators to monitor HAT elimination, backed by the Atlas of HAT, shows a robust and sustained trend towards the 2020 goal (i.e. elimination as public health problem), which appears well within reach. The positive epidemiological trend primarily owes to the efforts of NSSCP, supported by WHO and a range of committed stakeholders. To ensure the sustainability of these gains, and to pave the way for further progress, this support must be maintained. In this context, ownership of the elimination process and targets by endemic countries has to be reinforced, the development of improved tools has to continue, the availability of diagnostic tools must be ensured and WHO coordination of countries and other stakeholders must be ensured to maximize synergies. To this end, the WHO network for HAT elimination has been set up as the backbone for coordination and to facilitate the implementation of new tools and strategies. At this critical juncture in the process of elimination, the main challenge is to set up a cost-effective, adapted and sustained HAT control and surveillance strategies. This requires the progressive integration of HAT control activities in the general health system, which is often particularly difficult in those peripheral rural areas where the disease is more entrenched and the health system is weak. The sustainable elimination of HAT requires the reinforcement of the peripheral health system, a duty that falls beyond the HAT programmes and should be part of the Universal Health Coverage goal.

Whilst further decreasing the gambiense HAT burden seems possible, it will be important not to neglect the rhodesiense form, despite its relatively low number of cases. Unfortunately, as opposed to gambiense HAT, very few innovative tools have been developed for rhodesiense HAT screening, diagnosis and treatment. However, it is noteworthy that a clinical trial for the use of fexinidazole in rhodesiense HAT is now in preparation stage. The zoonotic nature of rhodesiense HAT does not presently allow to envisage the interruption of its transmission, whilst for the gambiense form, zero transmission increasingly appears as an attainable target.

Based on the data presented in this paper, and taking into account the epidemiological situation by country, the elimination of HAT as public health problem could be considered as already achieved in a number of HAT endemic countries. The criteria to assess and validate this status have been developed by WHO to enable the claim by countries and the formal recognition by WHO.

Beyond the elimination of HAT as public health problem, there is a pressing need to develop the strategies and intensify selected interventions towards the following target, the elimination as interruption of HAT transmission (zero cases) for the gambiense form of the disease. In this prospective scenario, adapted and cost-effective strategies are bound to rely more heavily on disease surveillance, and the sustained commitment of donors will be crucial. Also, clarifying the epidemiological role played by human asymptomatic carriers, by parasites in the skin, and by the possible animal reservoirs in gambiense HAT epidemiology, will be essential, as they may pose a threat to the full interruption of transmission, and to avert its resurgence or reintroduction [24, 25].

## Supporting information

S1 File Area at risk of gambiense and rhodesiense HAT. Period 2012–2016 (by country).
(DOCX)

S2 File Population at risk of gambiense and rhodesiense HAT. Period 2012–2016 (by country) and global trends from 2000–2004 to 2012–2016.
(DOCX)

S3 File Fixed health facilities offering different types of diagnosis and treatment of gambiense and rhodesiense HAT (by country). Data were collected by WHO from National Sleeping Sickness Control Programmes in June 2017).
(DOCX)

S4 File Geographic distribution of fixed health facilities offering different types of diagnosis and treatment of gambiense and rhodesiense HAT. Data were collected by WHO from National Sleeping Sickness Control Programmes in June 2017.
(DOCX)
