# Supplementary material for: Monitoring the elimination of human African trypanosomiasis: Update to 2016
Source: PLoS Negl Trop Dis. 2018 Dec 6;12(12):e0006890. doi: 10.1371/journal.pntd.0006890 (PMC6283345; doi:10.1371/journal.pntd.0006890)
Supplement: S1 File — Period 2012–2016 (by country). (DOCX) [file pntd.0006890.s001.docx]

# Area at risk of gambiense and rhodesiense HAT

Table A Areas at risk of *T. b. gambiense* infection (km^2^). Period 2012–2016.

| **Country** | **Total country area*** | **Area at risk**  **2012-2016** | | | | |
| --- | --- | --- | --- | --- | --- | --- |
|  |  | **Very High**  **and High** | **Moderate** | **Low and**  **Very Low** | **Total**  **at risk** | **% of total**  **country**  **area** |
| Angola | 1,253,770 | - | 3,855 | 84,160 | 88,015 | 7.0 |
| Burkina Faso | 274,470 | - | - | 1,307 | 1,307 | 0.5 |
| Cameroon | 466,396 | 208 | 1,261 | 8,117 | 9,585 | 2.1 |
| Central African Republic | 624,398 | 5,561 | 17,772 | 35,621 | 58,953 | 9.4 |
| Chad | 1,272,490 | 1,273 | 3,801 | 15,766 | 20,839 | 1.6 |
| Congo | 338,522 | 3,266 | 13,999 | 46,401 | 63,666 | 18.8 |
| Cote d'Ivôire | 321,363 | - | - | 8,799 | 8,799 | 2.7 |
| Democratic Republic of the Congo | 2,304,080 | 34,363 | 165,839 | 426,949 | 627,151 | 27.2 |
| Equatorial Guinea | 27,019 | - | 291 | 4,382 | 4,673 | 17.3 |
| Gabon | 265,978 | 396 | 4,605 | 7,685 | 12,686 | 4.8 |
| Ghana | 234,325 | - | - | 689 | 689 | 0.3 |
| Guinea | 246,094 | 134 | 2,242 | 8,511 | 10,887 | 4.4 |
| Nigeria | 908,866 | - | - | 466 | 466 | 0.1 |
| Sierra Leone | 72,777 | - | - | 1,060 | 1,060 | 1.5 |
| South Sudan | 633,356 | - | 8,889 | 61,947 | 70,836 | 11.2 |
| Uganda | 205,540 | - | 390 | 10,134 | 10,524 | 5.1 |
| Other Endemic Countries** | 2,954,255 | - | - | - | - | - |
| Total | 12,403,699 | 45,200 | 222,943 | 721,992 | 990,135 | 8.0 |

* Land area. The area of surface water bodies as depicted in the Shuttle Radar Topography Mission—River-Surface Water Bodies dataset is not included.

** Countries at marginal risk: Benin, Gambia, Guinea-Bissau, Liberia, Mali, Niger, Senegal and Togo.

Table B Areas at risk of *T. b. rhodesiense* infection (km^2^). Period 2012–2016.

| **Country** | **Total country area*** | **Area at risk**  **2012-2016** | | | | |
| --- | --- | --- | --- | --- | --- | --- |
|  |  | **Very High**  **and High** | **Moderate** | **Low and**  **Very Low** | **Total**  **at risk** | **% of total**  **country**  **area** |
| Kenya | 574,883 | - | - | 1,662 | 1,662 | 0.3 |
| Malawi | 94,758 | - | 1,961 | 12,834 | 14,795 | 15.6 |
| Mozambique | 779,061 | - | - | 480 | 480 | 0.1 |
| United Republic of Tanzania | 886,278 | - | 630 | 13,035 | 13,665 | 1.5 |
| Uganda | 205,540 | - | 904 | 10,690 | 11,593 | 5.6 |
| Zambia | 742,479 | 248 | 8,608 | 32,056 | 40,913 | 5.5 |
| Zimbabwe | 388,414 | - | 213 | 8,320 | 8,533 | 2.2 |
| Other Endemic Countries** | 2,593,564 | - | - | - | - | - |
| Total | 6,264,977 | 248 | 12,316 | 79,077 | 91,642 | 1.5 |

* Land area. The area of surface water bodies as depicted in the Shuttle Radar Topography Mission—River-Surface Water Bodies dataset is not included.

** Countries at marginal risk: Botswana, Burundi, Ethiopia, Namibia, Rwanda and Swaziland.
