# Supplementary material for: Monitoring the elimination of human African trypanosomiasis: Update to 2016
Source: PLoS Negl Trop Dis. 2018 Dec 6;12(12):e0006890. doi: 10.1371/journal.pntd.0006890 (PMC6283345; doi:10.1371/journal.pntd.0006890)
Supplement: S2 File — Period 2012–2016 (by country) and global trends from 2000–2004 to 2012–2016. (DOCX) [file pntd.0006890.s002.docx]

# Population at risk of gambiense and rhodesiense HAT

Table A Population at risk of *T. b. gambiense* infection (no. persons × 10^3^). Period 2012–2016.

| **Country** | **Total country population**  **2016*** | **Population at risk**  **2012-2016** | | | | | |
| --- | --- | --- | --- | --- | --- | --- | --- |
|  |  | **Very High**  **and High** | **Moderate** | **Low and**  **Very Low** | **Total**  **at risk** | **% of total**  **country**  **population** |  |
| Angola | 28,290 | - | 37 | 1,945 | 1,982 | 7.0 |  |
| Burkina Faso | 19,513 | - | - | 90 | 90 | 0.5 |  |
| Cameroon | 24,361 | - | 25 | 245 | 270 | 1.1 |  |
| Central African Republic | 5,507 | 40 | 217 | 409 | 666 | 12.1 |  |
| Chad | 11,852 | 24 | 148 | 696 | 868 | 7.3 |  |
| Congo | 4,852 | 14 | 63 | 2,513 | 2,590 | 53.4 |  |
| Cote d'Ivôire | 23,740 | - | - | 1,060 | 1,060 | 4.5 |  |
| Democratic Republic of the Congo | 81,331 | 669 | 6,576 | 30,224 | 37,469 | 46.1 |  |
| Equatorial Guinea | 759 | - | 2 | 42 | 44 | 5.9 |  |
| Gabon | 1,739 | 2 | 17 | 892 | 911 | 52.4 |  |
| Ghana | 26,908 | - | - | 58 | 58 | 0.2 |  |
| Guinea | 12,093 | - | 99 | 2,529 | 2,627 | 21.7 |  |
| Nigeria | 186,053 | - | - | 163 | 163 | 0.1 |  |
| Sierra Leone | 6,019 | - | - | 174 | 174 | 2.9 |  |
| South Sudan | 12,531 | - | 299 | 2,313 | 2,611 | 20.8 |  |
| Uganda | 38,319 | - | 10 | 1,540 | 1,550 | 4.0 |  |
| Other Endemic Countries** | 77,154 | - | - | - | - | - |  |
| Total | 561,022 | 750 | 7,494 | 44,891 | 53,134 | 9.5 |  |

* As per Landscan

** Countries at marginal risk: Benin, Gambia, Guinea-Bissau, Liberia, Mali, Niger, Senegal and Togo.

Table B Population at risk of *T. b. rhodesiense* infection (no. persons × 10^3^). Period 2012–2016.

| **Country** | **Total country population**  **2016*** | **Population at risk**  **2012-2016** | | | | |
| --- | --- | --- | --- | --- | --- | --- |
|  |  | **Very High**  **and High** | **Moderate** | **Low and**  **Very Low** | **Total**  **at risk** | **% of total**  **country**  **population** |
| Kenya | 46,791 | - | - | 28 | 27 | 0.1 |
| Malawi | 18,570 | - | 77 | 1,086 | 1,163 | 6.3 |
| Mozambique | 25,930 | - | - | 4 | 4 | 0.0 |
| United Republic of Tanzania | 52,483 | - | 3 | 303 | 306 | 0.6 |
| Uganda | 38,319 | - | 177 | 1,936 | 2,113 | 5.5 |
| Zambia | 15,511 | - | 25 | 457 | 482 | 3.1 |
| Zimbabwe | 13,601 | - | 4 | 123 | 127 | 0.9 |
| Other Endemic Countries** | 131,147 | - | - | - | - | - |
| Total | 342,351 | - | 286 | 3,935 | 4,222 | 1.2 |

* As per Landscan

** Countries at marginal risk: Botswana, Burundi, Ethiopia, Namibia, Rwanda and Swaziland.
