# Supplementary material for: Monitoring the elimination of human African trypanosomiasis: Update to 2016
Source: PLoS Negl Trop Dis. 2018 Dec 6;12(12):e0006890. doi: 10.1371/journal.pntd.0006890 (PMC6283345; doi:10.1371/journal.pntd.0006890)
Supplement: S3 File — Data were collected by WHO from National Sleeping Sickness Control Programmes in June 2017). (DOCX) [file pntd.0006890.s003.docx]

# Fixed Health Facilities for HAT

Table A Fixed Health Facilities for gambiense HAT: Survey March - June 2017. Differences to the survey September 2015 - February 2016 [9] in column ‘Δ’.

| **Country** | **Diagnosis** | | | | | | **Treatment** | | | | | | **TOTAL** | **Δ** |
| --- | --- | --- | --- | --- | --- | --- | --- | --- | --- | --- | --- | --- | --- | --- |
|  | **DxC** | **DxS** | **DxP** | **DxPh** | **Total Dx** | **Δ** | **Tx1P** | **Tx2M** | **Tx2E** | **Tx2N** | **Total Tx** | **Δ** |  |  |
| Angola | 60 | 60 | 27 | 20 | 60 | 41 | 20 | 0 | 17 | 12 | 20 | 3 | 60 | 41 |
| Benin | 3 | 3 | 0 | 0 | 3 | 0 | 0 | 0 | 0 | 0 | 0 | 0 | 3 | 0 |
| Burkina Faso | 8 | 8 | 2 | 2 | 8 | 1 | 2 | 2 | 2 | 0 | 2 | 1 | 8 | 1 |
| Cameroon | 12 | 5 | 9 | 8 | 14 | 3 | 10 | 0 | 6 | 6 | 10 | 0 | 14 | 3 |
| Central African Republic | 19 | 7 | 8 | 8 | 19 | 3 | 13 | 3 | 3 | 10 | 13 | 3 | 19 | 3 |
| Chad | 100 | 100 | 8 | 8 | 100 | 74 | 100 | 3 | 9 | 9 | 100 | 74 | 100 | 74 |
| Congo | 65 | 65 | 6 | 4 | 65 | 55 | 3 | 0 | 3 | 3 | 3 | -2 | 65 | 55 |
| Côte d’Ivoire | 4 | 4 | 1 | 1 | 4 | 0 | 1 | 1 | 1 | 1 | 1 | -3 | 4 | 0 |
| Democratic Republic of the Congo | 632 | 476 | 259 | 202 | 632 | 75 | 462 | 62 | 153 | 193 | 464 | 54 | 723 | 166 |
| Equatorial Guinea | 4 | 3 | 2 | 1 | 4 | 0 | 3 | 1 | 1 | 1 | 3 | 1 | 4 | 0 |
| Gabon | 5 | 5 | 1 | 1 | 5 | 1 | 1 | 0 | 1 | 1 | 1 | 0 | 6 | 2 |
| Ghana | 8 | 6 | 0 | 0 | 8 | 0 | 8 | 0 | 0 | 0 | 8 | 0 | 8 | 0 |
| Guinea | 115 | 115 | 3 | 3 | 115 | 104 | 3 | 2 | 0 | 3 | 3 | 0 | 115 | 104 |
| Mali | 6 | 6 | 3 | 0 | 8 | -3 | 0 | 0 | 0 | 0 | 0 | -1 | 8 | -3 |
| Nigeria | 50 | 50 | 5 | 5 | 50 | 0 | 5 | 0 | 0 | 5 | 5 | 0 | 50 | 0 |
| South Sudan | 15 | 5 | 8 | 5 | 15 | 0 | 5 | 5 | 5 | 5 | 5 | -4 | 15 | 0 |
| Togo | 2 | 2 | 0 | 0 | 2 | 0 | 0 | 0 | 0 | 0 | 0 | 0 | 2 | 0 |
| Uganda | 134 | 129 | 12 | 4 | 134 | 10 | 4 | 4 | 4 | 4 | 4 | 0 | 134 | 10 |
| Total | 1,242 | 1,049 | 354 | 272 | 1,246 | 364 | 640 | 83 | 205 | 253 | 642 | 126 | 1,338 | 456 |

DxC: clinical diagnosis; DxS: serological diagnosis; DxP: parasitological diagnosis; DxPh: disease staging. Tx1P: treatment of first-stage infection with pentamidine; Tx2M: treatment of second-stage infection with melarsoprol; Tx2E: treatment of second-stage infection with eflornithine; Tx2N: treatment of second-stage infection with nifurtimox-eflornithine combination therapy (NECT); Tx2: treatment of second-stage.

Δ: Difference between the survey March - June 2017 and the survey September 2015 - February 2016.

Table B Fixed Health Facilities for rhodesiense HAT: Survey March - June 2017. Differences to the survey September 2015 - February 2016 [9] in column ‘Δ’

| **Country** | **Diagnosis** | | | | | **Treatment** | | | | **TOTAL** | **Δ** |
| --- | --- | --- | --- | --- | --- | --- | --- | --- | --- | --- | --- |
|  | **DxC** | **DxP** | **DxPh** | **Total Dx** | **Δ** | **Tx1S** | **Tx2M** | **Total Tx** | **Δ** |  |  |
| Kenya | 25 | 22 | 16 | 25 | 0 | 15 | 15 | 15 | 14 | 25 | 0 |
| Malawi | 20 | 6 | 4 | 20 | 0 | 4 | 4 | 4 | 0 | 20 | 0 |
| Rwanda | 12 | 12 | 4 | 12 | 12 | 0 | 0 | 0 | 0 | 12 | 12 |
| Uganda | 37 | 36 | 12 | 37 | -1 | 10 | 10 | 10 | 3 | 37 | -1 |
| United Republic of Tanzania | 17 | 5 | 2 | 17 | 2 | 4 | 4 | 4 | -7 | 17 | 2 |
| Zambia | 12 | 12 | 12 | 12 | 0 | 10 | 10 | 10 | 2 | 12 | 0 |
| Zimbabwe | 1 | 1 | 1 | 1 | 0 | 1 | 1 | 1 | 0 | 1 | 0 |
| Total | 124 | 94 | 51 | 124 | 13 | 44 | 44 | 44 | 12 | 124 | 13 |

DxC: clinical diagnosis; DxP: parasitological diagnosis; DxPh: disease staging. Tx1S: treatment of first-stage infection with suramin; Tx2M: treatment of second-stage infection with melarsoprol.

Δ: Difference between the survey March - June 2017 and the survey September 2015 - February 2016.
