# Supplementary material for: Monitoring the elimination of human African trypanosomiasis: Update to 2016
Source: PLoS Negl Trop Dis. 2018 Dec 6;12(12):e0006890. doi: 10.1371/journal.pntd.0006890 (PMC6283345; doi:10.1371/journal.pntd.0006890)
Supplement: S4 File — Data were collected by WHO from National Sleeping Sickness Control Programmes in June 2017. (DOCX) [file pntd.0006890.s004.docx]

**Additional file**

**Geographic distribution of fixed health facilities having capacities for diagnosis of human African trypanosomiasis (March and June 2017)**
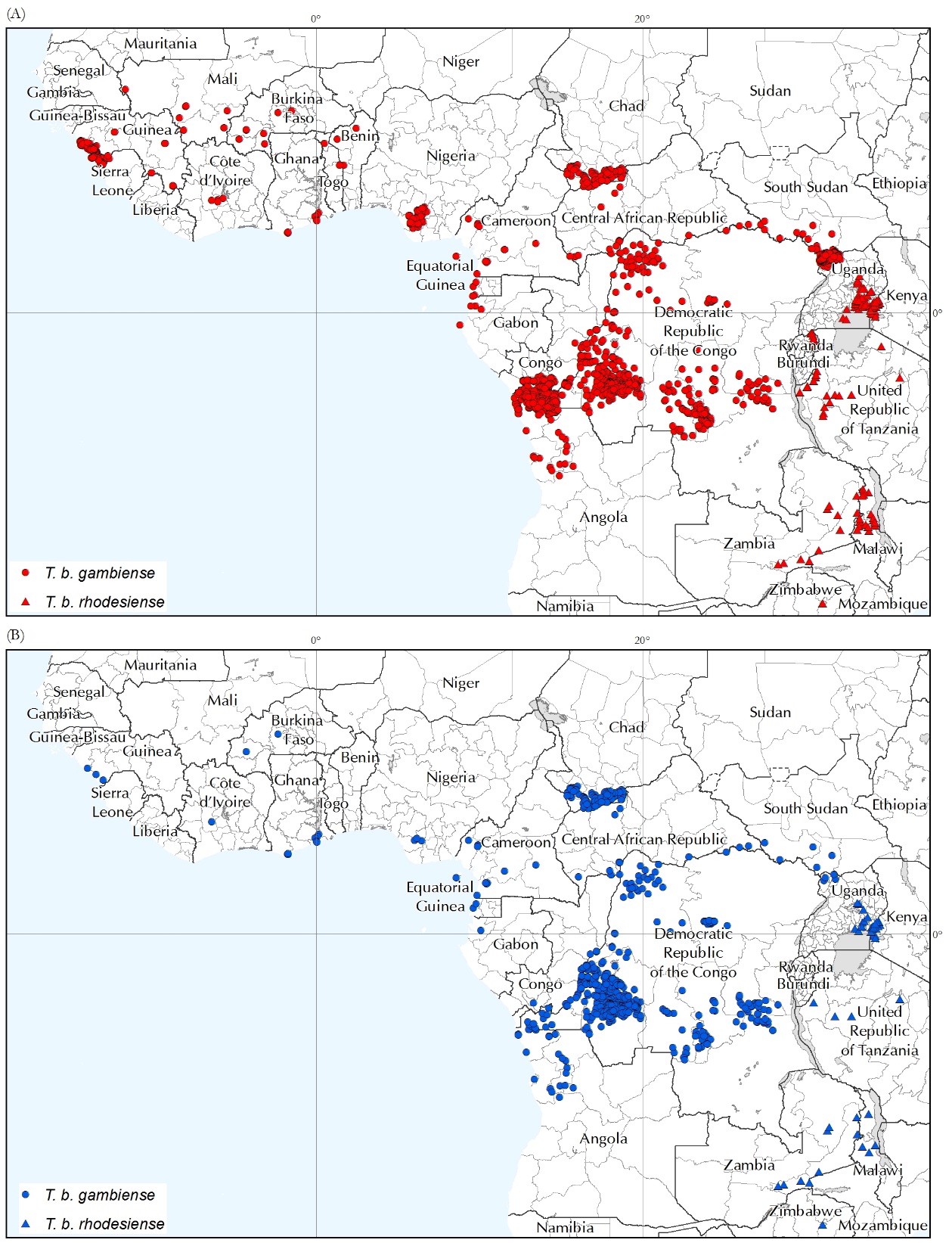


Figure A Geographic distribution of fixed health facilities having capacities for diagnosis (A) and treatment (B) of gambiense and rhodesiense HAT


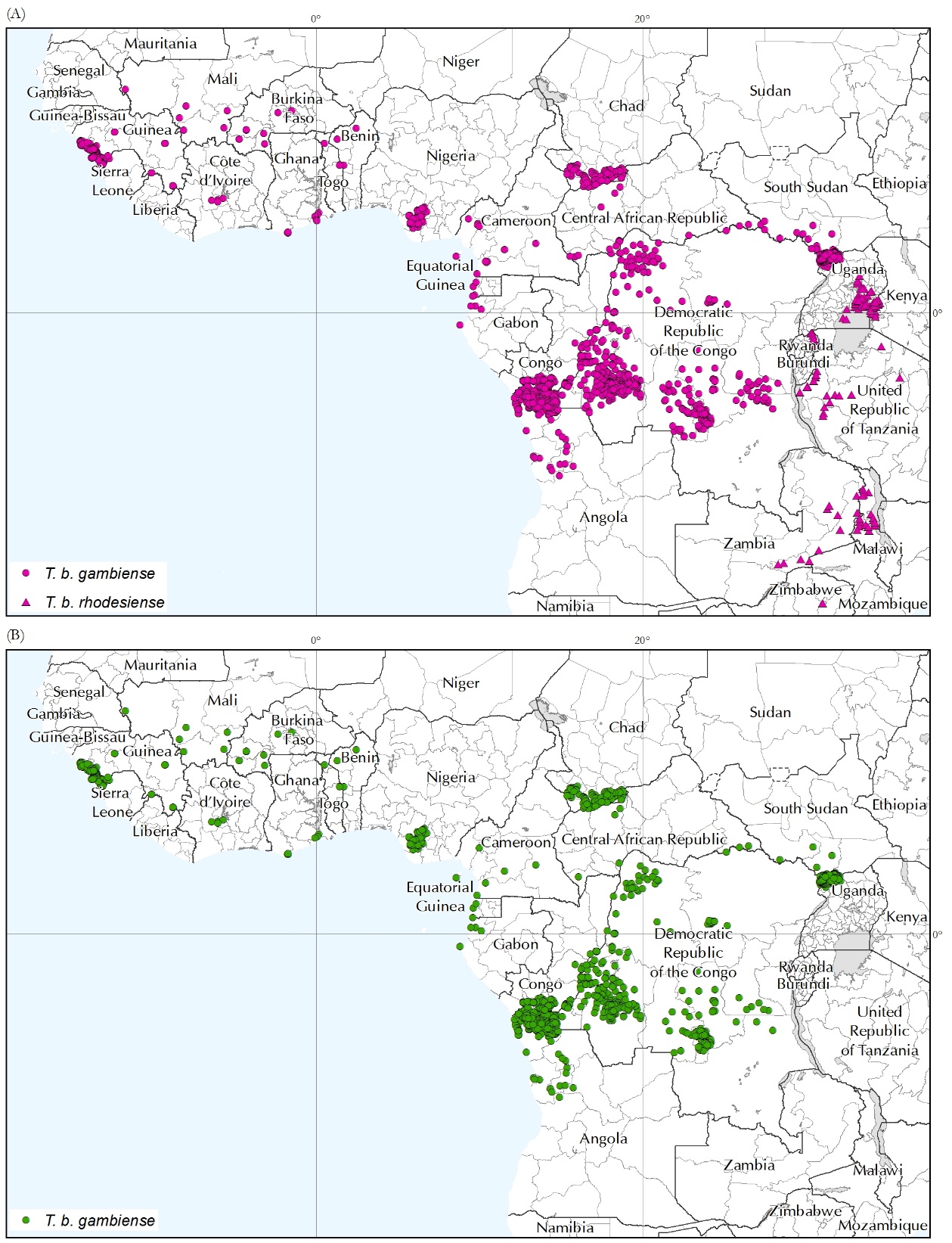


Figure B Geographic distribution of fixed health facilities having capacities for clinical diagnosis of gambiense and rhodesiense HAT (A) and serological diagnosis of gambiense HAT (B)


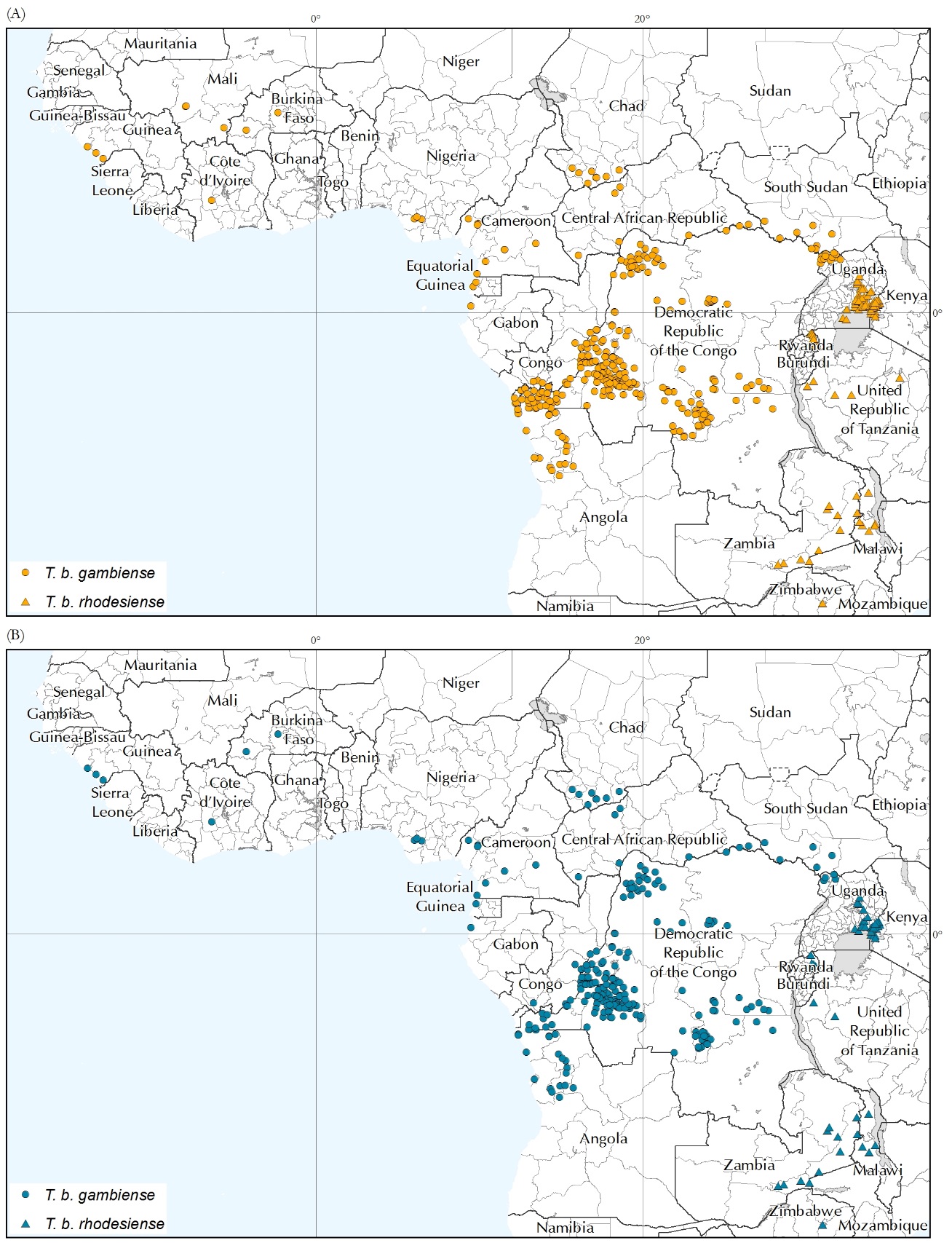


Figure C Geographic distribution of fixed health facilities having capacities for parasitological diagnosis of HAT (A) and stage determination (B)

**Geographic distribution of fixed health facilities having capacities for treatment of human African trypanosomiasis**
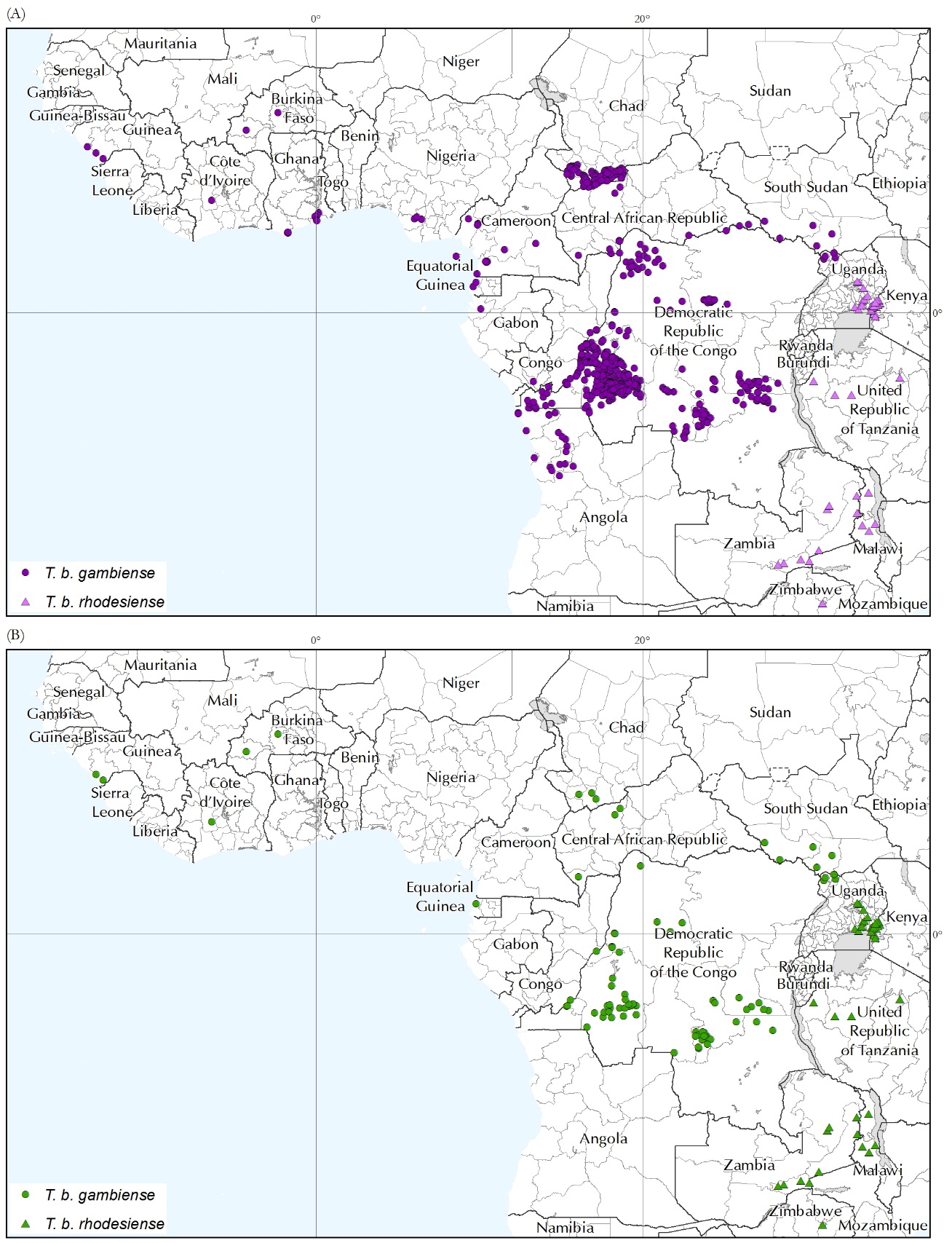


Figure D Geographic distribution of fixed health facilities having capacities for treatment of gambiense HAT first-stage infections with pentamidine and of rhodesiense HAT first-stage infections with suramin (A) and second-stage infection with melarsoprol (B)


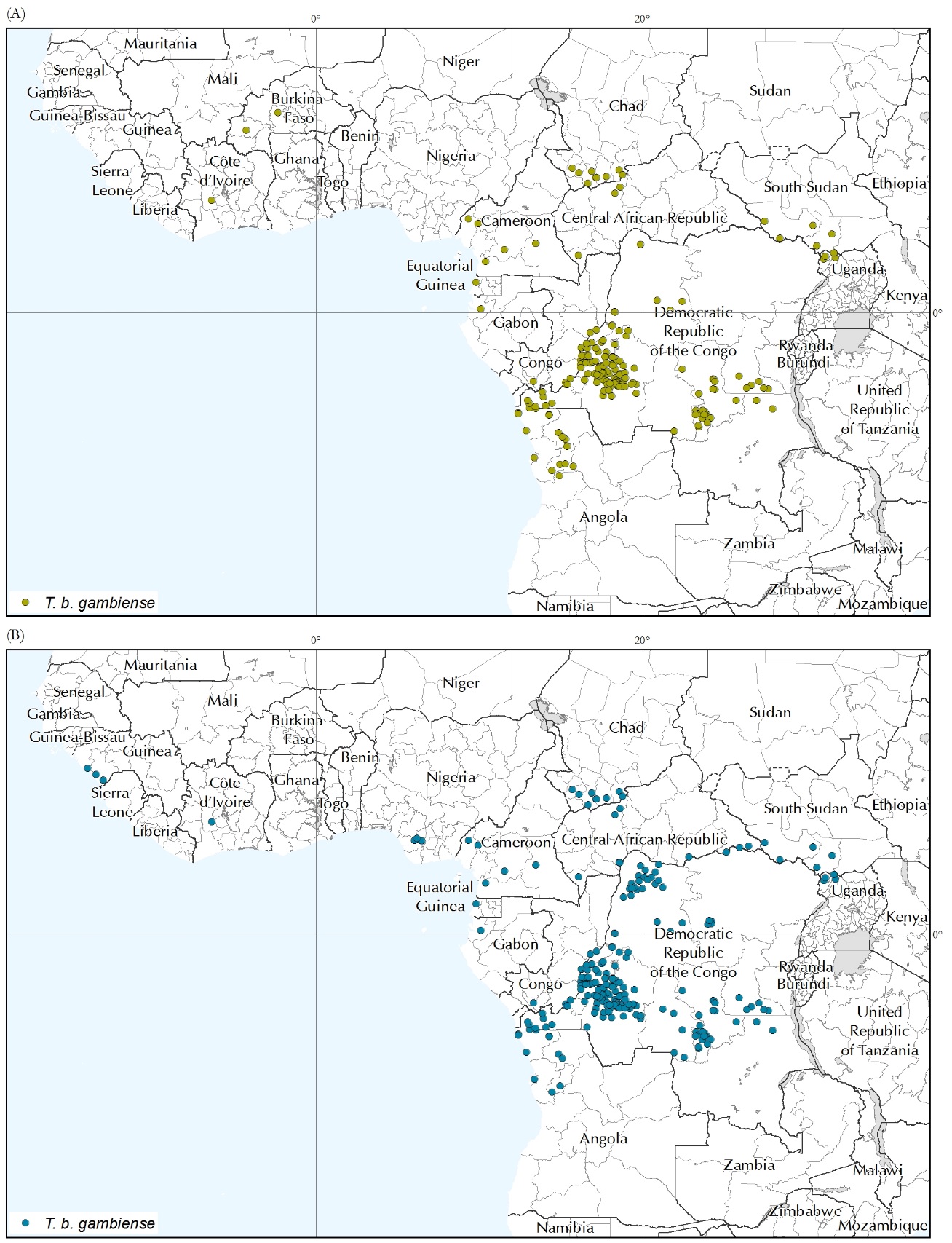


Figure E Geographic distribution of fixed health facilities having capacities for treatment of gambiense HAT second-stage infections with eflornithine (A) and with nifurtimox-eflornithine combination therapy (B)
